# Fractional Anisotropy of the Fornix and Hippocampal Atrophy in Alzheimer’s Disease

**DOI:** 10.3389/fnagi.2014.00316

**Published:** 2014-11-13

**Authors:** Kejal Kantarci

**Affiliations:** ^1^Department of Radiology, Mayo Clinic, Rochester, MN, USA

**Keywords:** fornix, DTI, Alzheimer’s disease, hippocampus, MRI imaging

## Abstract

Decrease in the directionality of water diffusion measured with fractional anisotropy (FA) on diffusion tensor imaging has been linked to loss of myelin and axons in the white matter. Fornix FA is consistently decreased in patients with mild cognitive impairment (MCI) and Alzheimer’s disease (AD). Furthermore, decreased fornix FA is one of the earliest MRI abnormalities observed in cognitively normal individuals who are at an increased risk for AD, such as in pre-symptomatic carriers of familial AD mutations and in pre-clinical AD. Reductions of FA at these early stages, which predicted the decline in memory function. Fornix carries the efferent projections from the CA1 and CA3 pyramidal neurons of the hippocampus and subiculum, connecting these structures to the septal nuclei, anterior thalamic nucleus, mammillary bodies, and medial hypothalamus. Fornix also carries the afferent cholinergic and GABAergic projections from the medial septal nuclei and the adjacent diagonal band back to the medial temporal lobe, interconnecting the core limbic structures. Because fornix carries the axons projecting from the hippocampus, integrity of the fornix is in-part linked to the integrity of the hippocampus. In keeping with that, fornix FA is reduced in subjects with hippocampal atrophy, correlating with memory function. The literature on FA reductions in the fornix in the clinical spectrum of AD from pre-symptomatic carriers of familial AD mutations to pre-clinical AD, MCI, and dementia stages is reviewed.

## Anatomy of the Hippocampus and Fornix Connections

Hippocampal projections to the subcortical structures that form the fimbria are carried by the fornix bundle. The fornix has two major components that connect hippocampus and subcortical structures. These are the larger postcommissural component and the smaller precommissural component. A majority of the axonal projections in the fornix originate from the subiculum of the hippocampus and form the postcommissural fornix carrying subicular axons to the mammillary bodies, which then project to the anterior nucleus of the thalamus. A major axonal tract originating from the anterior thalamus projects to the posterior cingulate cortex, which connects back to hippocampus via the cingulum tract closing the “Papez Circuit” (Benarroch, 2006). In addition, the precommissural fornix caries axons originating from the CA1 and CA3 pyramidal neurons of the hippocampus to the lateral septal nucleus, which projects to the medial septum. The medial septum and the nucleus of the diagonal band send cholinergic and GABAergic projections back to the medial temporal lobe via the fornix.

Damage to fornix in monkeys produces deficits in learning about the places of objects and about the places where responses should be made. They are impaired in using information about their place in an environment. Furthermore, fornix lesions impair conditional left–right discrimination learning based on visual appearance of an object on either side (Rolls, 2014). Fornix transection in human beings has been reported to produce significant and persistent anterograde amnesia (D’Esposito et al., 1995).

Because majority of the axonal projections carried by the fornix originate from the subiculum and to a lesser extent from CA1 and CA3 pyramidal neurons of the hippocampus, neurodegenerative processes impacting these neuronal populations would also lead to degeneration of the axonal projections originating from these neurons. However, it is important to note that not all axons in the fornix originate from the hippocampus, as the cholinergic and GABAergic connections originating from the medial septum and the nucleus of the diagonal band project back to the medial temporal lobe via the fornix. Therefore, degeneration of neuronal populations in the hippocampus may not fully explain the axonal loss in the fornix.

## Normal Aging and the Hippocampus–Fornix Axis

In monkeys, number of myelinated nerve fibers in the fornix decrease with aging by 15–34%, but the loss of myelinated nerve fibers in the fornix is not disproportionately high or low compared to the rest of the white matter. However, it is not clear whether the myelinated nerve fiber loss from the fornix with age is due to loss of the parent neurons from the hippocampus, or due to age-related alterations that affect myelin sheaths, and alter the conduction velocities along nerve fibers resulting in degeneration in long projecting myelinated nerve fiber tracts. This loss of nerve fibers lead to a reduced subcortical connectivity of the hippocampus and may in-part be responsible for cognitive decline associated with normal aging even when the hippocampal neurons are intact (Peters et al., 2010).

Aging is associated with alterations in hippocampal dendritic morphology in the absence of neuronal loss, therefore age-associated structural alterations in the fornix less likely to originate from hippocampal neuronal loss, and more likely be the consequence of demyelination and degeneration of fornix fibers with age (Hof and Morrison, 2004). While both hippocampal volumes (Jack et al., 1998), and white matter fractional anisotropy (FA) decreases during aging (Walhovd et al., 2010), a relationship between fornix FA and hippocampal volume remains even after controlling for age. Using a recursive regression procedure, to evaluate sequential relationships between the alterations of the hippocampus and fornix, Pelletier et al. showed that hippocampal atrophy in healthy aging could be mediated by a loss of fornix connections potentially through a retrograde process (Pelletier et al., 2013). Longitudinal change in hippocampal volumes and fornix FA over the adult lifespan may be useful in determining whether hippocampal volume loss or a decrease in fornix FA occur earlier during the aging process.

Sex differences in structural network connectivity across the life span have been observed in DTI studies, with women showing greater overall cortical connectivity and network organization and greater efficiency than men (Gong et al., 2009). However, it is unclear at this time whether there is a difference in structural connectivity across the hippocampus-fornix axis among men and women.

## Hippocampus-Fornix Axis in Prodromal and Pre-Clinical AD

There is converging evidence from longitudinal studies on clinically followed and autopsied cohorts that most people with the amnestic form of mild cognitive impairment (MCI) who progress to dementia in the future, develop Alzheimer’s disease (AD) (Flicker et al., 1991; Bowen et al., 1997; Morris et al., 2001; Bennett et al., 2002; Meyer et al., 2002; Jicha et al., 2006; Petersen et al., 2006). In keeping with these findings, both hippocampal atrophy and a reduction in fornix FA are frequently observed in amnestic MCI (Liu et al., 2011; Zhuang et al., 2012, 2013; Zhang et al., 2013) and are associated with memory decline (Mielke et al., 2012) and the risk of progression to AD (Mielke et al., 2012; Douaud et al., 2013).

**Figure 1:**
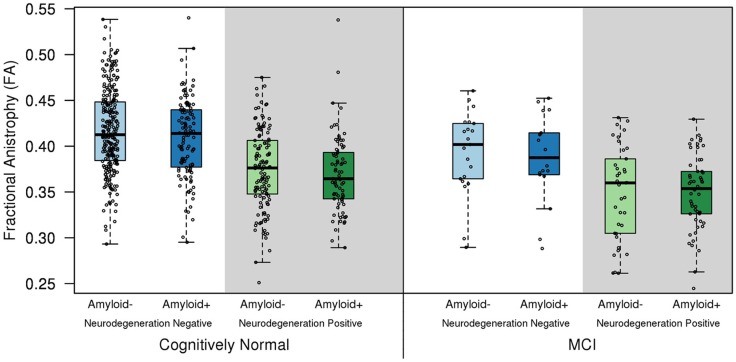
**Fractional anisotropy of the fornix in the cognitively normal (CN) and mild cognitive impairment (MCI) biomarker groups**. Hippocampal atrophy on MRI and/or hypometabolism in the Alzheimer signature composite on FDG PET was used to classify subjects into the neurodegeneration-positive group, and high amyloid load on PET was used to classify subjects into the amyloid-positive group. Cut-points for amyloid positivity, hippocampal atrophy, and Alzheimer signature hypometabolism were determined from the 10th percentile of the measurement distributions in clinically diagnosed AD patients as previously described (Jack et al., 2012). Fornix FA was lower in the neurodegeneration positive CN and MCI patients compared to the cognitively normal group with normal imaging findings (amyloid and neurodegeneration negative; *p* < 0.001). A positive amyloid PET scan was not associated with a reduction in fornix FA, in the absence of co-existent neurodegeneration in cognitively normal individuals (*p* = 0.19) (Kantarci et al., 2014).

The relationship between the structural integrity of hippocampus and fornix has been demonstrated in MCI (Mielke et al., 2012; Zhuang et al., 2012). In a cohort of cognitively normal (CN) older adults (*n* = 570; median age = 78; interquartile range = 74-83) and MCI (*n* = 131; median age = 80; interquartile range = 77–86) from the community, both CN and MCI patients showed decreased fornix FA if they also had hippocampal atrophy or AD pattern of hypometabolism on ^18^F Fluorodeoxyglucose (FDG) PET compared to the CN group with normal hippocampal volumes and metabolism on FDG PET. Having only a positive amyloid-β PET scan with Pittsburgh compound-B (PiB) was not responsible for a reduction in fornix FA in CN individuals, demonstrating that high amyloid load does not influence diffusion tensor imaging (DTI)-based measures of white matter integrity in the absence of co-existent gray matter neurodegeneration in non-demented older adults (Figure 1) (Kantarci et al., 2014). Although individuals with MCI and pre-clinical AD have reductions in fornix FA, it should be noted that a reduction in fornix FA by itself would be insufficient in classifying preclinical and prodromal AD. However, fornix FA can be combined with other imaging biomarkers such as hypometabolism on FDG PET, hippocampal atrophy, and amyloid-β PET for a more accurate classification of individuals at risk for AD, and predicting outcomes.

In carriers of the fully penetrant familial AD mutations (Ringman et al., 2007), a reduction in fornix FA was present in CN individuals who were destined to develop AD regardless of the cross-sectional area of the fornix, suggesting that the FA reduction in the fornix may precede hippocampal atrophy and clinical symptoms in AD. It is not possible to make inferences on whether FA reductions precede hippocampal atrophy on MRI in the course of AD based on findings from cross-sectional studies. Longitudinal investigations with serial DTI and structural MRI in pre-clinical AD and MCI may clarify the sequence of DTI and volumetric MRI findings in AD.

## Hippocampus-Fornix Axis in Alzheimer’s Disease

There is a topographic concordance between gray and white matter diffusivity changes in AD (Kantarci et al., 2010), which implies that the disruption in white matter tracts is closely associated with the cortical pathology. Late-myelinating fibers in the brain such as the corticocortical association and the limbic pathways that are more vulnerable to degeneration related to AD than the early-myelinating fibers such as the corticospinal tracts, which do not show any DTI abnormalities in early AD (Stricker et al., 2009; Kantarci et al., 2010; Oishi et al., 2012).

Consistent with the findings in normal aging and MCI and pre-clinical AD, correlations between fornix FA and hippocampal atrophy has been observed in AD (Firbank et al., 2007; Pievani et al., 2010; Lee et al., 2012). In a sub-regional analysis of the hippocampus Lee et al. reported that the strongest associations between the structural changes in the hippocampus and fornix diffusivity is localized to the hippocampal CA1 and anteromedial subiculum (Lee et al., 2012). CA1 and subiculum are the sub-regions of the hippocampus that are vulnerable to AD-related neurodegeneration (Davies et al., 1992). Hippocampal efferent fibers that pass through the fornix are thought to originate from both of these subfields. The authors concluded that: “Cortical neuronal damage and subcortical axonal defects in AD are likely to be closely linked with each other, possibly reflecting a suggested pathogenic interaction between the two” (Lee et al., 2012). However, they also acknowledged that this hypothesis needs to be tested through longitudinal studies.

## Conclusion

The association of microstructural alterations in the hippocampus and fornix continues throughout human lifespan and remains even when these structures are impacted by AD starting from the preclinical and prodromal stages. Prospective studies investigating the rates of structural alterations in these two structures may clarify the temporal sequence of the reductions in fornix FA and hippocampal volumes through preclinical, prodromal, and dementia stages of AD.

## Conflict of Interest Statement

The author declares that the research was conducted in the absence of any commercial or financial relationships that could be construed as a potential conflict of interest.
